# Mouse models, antibodies, and neuroimaging: Current knowledge and future perspectives in neuropsychiatric systemic lupus erythematosus (NPSLE)

**DOI:** 10.3389/fpsyt.2023.1078607

**Published:** 2023-03-08

**Authors:** Vanessa Tomalla, Michael J. Schmeisser, Julia Weinmann-Menke

**Affiliations:** ^1^Department of Internal Medicine, Division of Nephrology, University Medical Center of the Johannes Gutenberg University Mainz, Mainz, Germany; ^2^Institute of Anatomy, University Medical Center of the Johannes Gutenberg University Mainz, Mainz, Germany; ^3^Focus Program Translational Neurosciences (FTN), University Medical Center of the Johannes Gutenberg University Mainz, Mainz, Germany; ^4^Research Center for Immunotherapy (FZI), University Medical Center of the Johannes Gutenberg University Mainz, Mainz, Germany

**Keywords:** systemic lupus erythematosus, neuropsychiatric systemic lupus erythematosus, autoimmune disease, anti-N-Methyl-D-Aspartic Acid Receptor 2 antibodies, mouse models, autoantibodies, NMDA-receptor, neuroimaging

## Abstract

As a chronic autoimmune disease systemic lupus erythematosus (SLE) can also affect the central and the peripheral nervous system causing symptoms which are summed up as neuropsychiatric systemic lupus erythematosus (NPSLE). These symptoms are heterogenous including cognitive impairment, seizures, and fatigue, leading to morbidity or even mortality. At present, little is known about the pathophysiological processes involved in NPSLE. This review focuses on the current knowledge of the pathogenesis of NPSLE gained from the investigation of animal models, autoantibodies, and neuroimaging techniques. The antibodies investigated the most are anti-ribosomal P protein antibodies (Anti-rib P) and anti-N-Methyl-D-Aspartic Acid Receptor 2 antibodies (Anti-NR2), which represent a subpopulation of anti-dsDNA autoantibodies. Experimental data demonstrates that Anti-rib P and Anti-NR2 cause different neurological pathologies when applied intravenously (i.v.), intrathecally or intracerebrally in mice. Moreover, the investigation of lupus-prone mice, such as the MRL/MpJ-Fas*^lpr/lpr^* strain (MRL/lpr) and the New Zealand black/New Zealand white mice (NZB × NZW F1) showed that circulating systemic antibodies cause different neuropsychiatric symptoms compared to intrathecally produced antibodies. Furthermore, neuroimaging techniques including magnetic resonance imaging (MRI) and positron emission tomography (PET) are commonly used tools to investigate structural and functional abnormalities in NPSLE patients. Current research suggests that the pathogenesis of NPSLE is heterogenous, complex and not yet fully understood. However, it demonstrates that further investigation is needed to develop individual therapy in NPSLE.

## Introduction

Systemic Lupus erythematosus (SLE) is a chronic autoimmune disease, which attacks multiple organs and tissues, such as the renal or the mucocutaneous system (1). It is characterized by antinuclear antibodies and results in heterogeneous clinical manifestations (2). There are various separate syndromes affecting the central and the peripheral nervous system in SLE, which are all summed up as neuropsychiatric systemic lupus erythematosus (NPSLE) (3). Depending on the study design the prevalence of NPSLE ranges from 6 to 91% (4–6).

According to the American College of Rheumatology (ACR), NPSLE comprises 19 neuropsychiatric manifestations, that can be focal or diffuse, and vary from subtle cognitive dysfunction to severe acute diseases such as seizure disorders, demyelinating syndromes, and psychosis (7). Among 12 syndromes of the central nervous system (CNS) headache, anxiety disorder, seizure, and cognitive disfunction represent the most common (8). Further, the ACR named seven syndromes of the peripheral nervous system (PNS), including poly- and mononeuropathy (7). These neuropsychiatric symptoms represent one of the main reasons for a decreased quality of life in SLE (9). Not treated sufficiently, NPSLE can lead to severe morbidity or even mortality (10).

Up to now, little is known about the pathophysiological processes causing different patterns of neuropsychiatric involvement in SLE. However, several studies demonstrate an important role of autoantibodies as well as the disruption of the blood brain barrier (BBB) (11, 12). Moreover, established mouse models and neuroimaging methods prove themselves as key tools to investigate the pathomechanisms of NPSLE (13, 14).

This review highlights the current knowledge of the pathogenesis of NPSLE gained from the investigation of animal models, antibodies, and neuroimaging techniques.

## Mouse models

### MRL/MpJ-Fas*^lpr/lpr^*

One of the most frequently used mouse models is the MRL/MpJ-Fas*^lpr/lpr^* strain (MRL/lpr), which spontaneously develops an SLE phenotype, including serological markers and behavioral dysfunction. The LPR gene leads to a loss of Fas function resulting in a longer survival of autoreactive lymphocytes and thus higher autoantibody titers (15). MRL/lpr mice develop neuropsychiatric symptoms at an early stage of disease (approx. 8 weeks of age), even when yet no other organ is affected (16).

The symptoms MRL/lpr display the most frequently are depression (including lack of motivation, learned helplessness, fatigue, and apathy) and impaired cognition (especially learning and spatial memory) (14). It is shown, that the severity of depression correlates with the presence and titers of autoantibodies against nuclear antigens, N-Methyl-D-Aspartic Acid (NMDA-) receptors, and ribosomal P proteins in MRL/lpr mice as well as in human patients (16, 17). An adoptive transfer study performed by Katzav et al. showed that intracerebroventricularly injected anti-ribosomal P protein antibodies (anti-rib P) from patients with SLE were capable of inducing autoimmune depression in healthy mice (18).

There is evidence that a main reason for the development of NPSLE in human is the presence of pathogenic autoantibodies in the cerebrospinal fluid (CSF) and in the brain parenchyma, respectively. But how do these cells and antibodies trespass the BBB? Besides the fact that circulating systemic antibodies can enter the CNS after the disruption of the BBB, experimental studies show that MRL/lpr mice also produce antibodies intrathecally (19, 20). Circulating antibodies and intrathecally produced antibodies cause different neuropsychiatric symptoms. Experimental studies show that intrathecally generated antibodies in MRL/lpr mice correlate with increased depressive-like behavior while anxiety-like behavior is rather associated with circulating antibodies (20, 21). Further, complement activation plays a pivotal role in the development of neuroinflammation and neurodegeneration in the brains of MRL/lpr mice (14, 22). The effect of complement activation can be apoptosis, the upregulation of proinflammatory cytokines as well as an increased permeability of the BBB (19, 23).

Briefly, MRL/lpr mice have already unraveled possible pathogenetic pathways in the development of NPSLE, such as the involvement of autoantibodies, inflammation and cytokines, and the pathologies of the BBB.

### New Zealand black/New Zealand white

The New Zealand black/New Zealand white mice (NZB × NZW F1) also spontaneously develop a lupus-like autoimmune disease with high titers of anti-nuclear antibodies. There are various complex genetic mutations, including MHC class II polymorphisms that are found to be involved in the development of SLE (24, 25). Besides, a dysregulation of T-helper cell cytokines and complex intrinsic B-cell defects seem to play a role in the pathogenesis of SLE in the NZB x NZW F1 strain (26). NZB × NZW F1 mice develop a systemic disease and serological markers resembling human SLE, such as high titers of anti-nuclear and anti-double-stranded DNA (dsDNA) antibodies (27).

These autoimmune mice also exhibit neuropsychiatric behavior but in comparison to MRL/lpr mice at an older age and different phenotype (28). The neurobehavioral symptoms in NZB × NZW F1 include decreased postural response and righting reflexes, impaired learning and memory as well as increased anxiety (28, 29).

### Other mouse models

There are numerous other mouse models that spontaneously develop an autoimmune disease, such as the BXSB/Yaa, the 564Igi, and the C57Bl/6-Fas*^lpr/lpr^* strain (30–32). These models exhibit different characteristics of SLE and are generally used to investigate more specific questions.

Besides spontaneous SLE mice, there are also induced mouse models, in which the mice develop autoimmune disease after being exposed to specific agents. One example is the injection of pristane or the immunization of mice with a peptide mimotope of DNA (DWEYS) (33, 34). Whereas the spontaneous models of SLE provide an important insight into the complex genetics of NSPLE, induced mouse models are used to evaluate the impact of environmental factors triggering the disease in a genetically prone individual (35).

## Autoantibodies in NPSLE

A main characteristic of SLE is the presence of antibodies that bind to self-antigens and thus initiate an autoimmune reaction. The new SLE classification criteria, developed with the support by both the European League Against Rheumatism (EULAR) and the ACR, defined antinuclear antibodies (titer ≥ 1:80) as an entry criterion for the diagnosis of SLE (2). The development of autoimmune antibodies and the formation of immune complexes mediate the pathogenesis of SLE by triggering the activation of both the innate and the adaptive immune system. This results in complement deposition and infiltration of immune cells causing inflammation and damage in the affected organs (36).

### Anti-N-Methyl-D-Aspartic Acid Receptor 2 (Anti-NR2), a subpopulation of anti-dsDNA autoantibodies

The NMDA-receptor is a ionotropic glutamate receptor in the CNS, which is indispensable for synaptic plasticity and memory (37). NMDA-receptors are hetero-oligomers which encompass two NR1 (or GluN1) subunits and two out of four existing NR2 (or GluN2) (A–D) subunits (38). The receptor is physiologically activated by the binding of glutamate to the NR2 subunit and glycine to the NR1 subunit, which results in a voltage-dependent excitatory influx of Na^+^-ions and Ca^2+^-ions into the cell (39).

**FIGURE 1:**
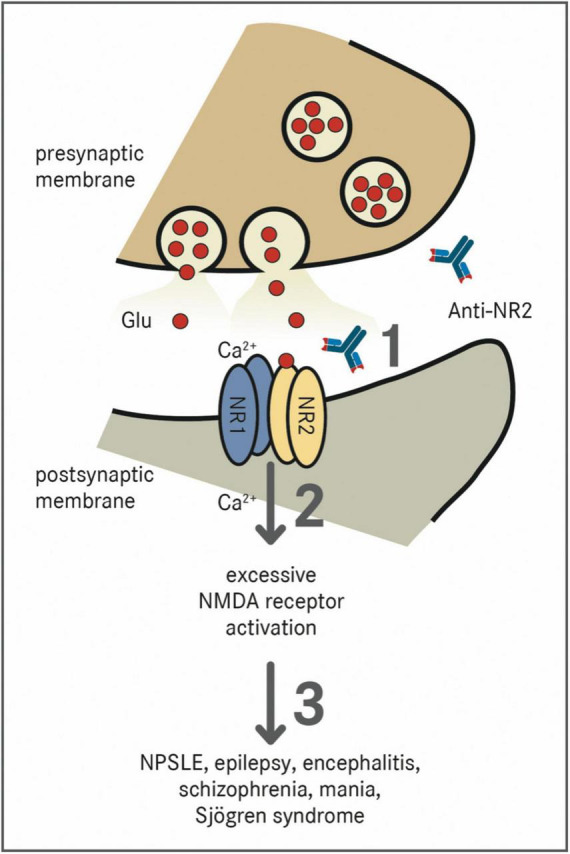
Effect of Anti-NR2 on NMDA receptors. NMDA receptors are tetramers which are composed of two NR1 subunits and two out of four NR2 (A–D) subunits (38). The receptor is physiologically activated by the binding of glutamate (Glu) to the NR2 subunit and glycine to the NR1 subunit, which results in a voltage-dependent excitatory influx of Na^+^-ions and Ca^2+^-ions into the cell (39). (1) Pathologically, Anti-NR2 antibodies (Anti-NR2) bind to the extracellular domains of the NR2A and/or NR2B subunit of the NMDA receptor (40). (2) These antibodies act as positive allosteric modulators with a much higher sensitivity to the NR2A subunit leading to an excessive activation of the NMDA receptor (42). (3) The presence of Anti-NR2 is associated with the diseases such as NPSLE, epilepsy, encephalitis, schizophrenia, mania, or Sjögren syndrome (40).

Antibodies against the NR2 subunit (Anti-NR2) can be found in patients suffering from SLE, epilepsy, encephalitis, schizophrenia, mania, stroke, or Sjögren syndrome (40). In NPSLE, there are antibodies directed against NR2A and NR2B, which are a subpopulation of anti-dsDNA autoantibodies cross-reacting with the respective NMDAR subunits (40). Anti-NR2 antibodies bind to the *N*-terminal domains of the NR2A and NR2B subunit of the NMDA-receptor containing the Asp/Glu-Trp-Asp/Glu-Tyr-Ser/Gly (DWEYS) pentapeptide sequence (41). The consensus sequence is present in dsDNA as well as the extracellular domain of the NR2A and NR2B subunits. Anti-NR2 act as positive allosteric modulators with a much higher sensitivity to NR2A than to NR2B (42). Figure 1 schematically shows the pathomechanism of Anti-NR2.

In NPSLE, autoantibodies in the serum do not automatically reflect their presence in the CSF (43). The exact percentage of SLE patients having Anti-NR2 varies in different studies from 14 to 35% (44, 45). When found in the CSF, Anti-NR2 are closely related to diffuse NPSLE, causing symptoms such as depression and cognitive dysfunction (46). Schwarting et al. analyzed a large SLE cohort and revealed that the Anti-NR2 concentration in the serum correlated with the severity and the incidence of fatigue in these patients (47). Whereas the group’s *in vitro* studies showed a (maybe reversible) reduced ATP metabolism in astrocytes, other experimental studies claim neuronal excitotoxicity as a main pathomechanism of Anti-NR2. It is believed that death of neurons is primarily due to increased calcium influx which results in the induction of apoptosis (41, 48). These different results indicate that both the concentration of antibodies and the duration of exposure may play a role in antibody pathogenicity and might lead to different clinical neuropsychiatric manifestations caused by the same antibody.

The NR2A and NR2B subunits are expressed the highest in the hippocampus, the amygdala, and the hypothalamus (40). A decreased hippocampal volume can be found in patients with fatigue and circulating Anti-NR2 over a time period of 2 years, suggesting a structural effect of a long-time exposure to these autoantibodies (47).

When Anti-NR2 is applied to mice *in vivo*, neuropathological changes were only observed when injected into brains or after pharmacological disruption of the BBB (41, 49, 50). Up to now, it remains uncertain whether the transfer of systemic Anti-NR2 to the CNS is obligatory or if these autoantibodies can also be produced intrathecally. However, there are studies implicating further pathogenic effects of Anti-NR2 involving microglia, cytokines, and T-cells (51, 52).

### Anti-ribosomal P protein antibodies (Anti-rib P)

Anti-rib P are directed against the carboxy-terminal regions of three ribosomal P proteins: P1, P2, and P0 (53). There are different pathomechanisms hypothesized concerning the involvement of anti-rib P in NPSLE. One states that these antibodies cross-react with the neuronal surface P antigen, which is especially found in the membrane of neurons of the hippocampus and thus impair its function, which clinically manifests as depression (54, 55). It is also known, that Anti-rib P cross-react with NMDA-receptors causing diffuse neuropsychiatric symptoms, such as psychosis, in NPSLE (18, 56).

There are indications, that the neuropathological impact of Anti-rib P antibodies is caused by neuron cell death and impaired synaptic plasticity (57).

### Other antibodies

**Anti-phospholipid antibodies (aPL).** The most frequently investigated aPL in SLE are anticardiolipin, anti-β2-glycoprotein 1 antibodies, and lupus anticoagulant. They bind to phospholipids and associated proteins in the plasma membrane and activate endothelial cells, platelets, and monocytes causing a hypercoagulable state (58). Presence of aPL antibodies primarily results in thrombosis and cerebral infarction, thereby leading to neuropsychatric symptoms in SLE (59). Moreover, there are symptoms in patients with aPL, including seizures, chorea, and cognitive dysfunction, suggesting, that these manifestations are not due to thrombotic events, but underlie a different pathogenic mechanism (60).

**Anti-endothelial cell antibodies** are found more often in SLE patients with neuropsychiatric symptoms than without (61). They have a proinflammatory effect on endothelial cells, which results in an enhanced expression of adhesion molecules and in the secretion of cytokines. This promotes rolling and adhesion of leucocytes to the endothelium of the BBB and finally the diapedesis into the brain parenchyma (62). These antibodies primarily seem to be involved in the disruption of the BBB.

**Anti-microtubule-associated protein 2 antibodies** represent one of the brain-specific antibodies. This protein is expressed in neurons and is involved in the stabilization of microtubules and is associated with psychosis and seizure in NPSLE (63, 64).

## Neuroimmune interfaces and the cerebrospinal fluid

The brain contains four fluid compartments including the cerebrospinal fluid (CSF), interstitial fluid, intracellular fluid, and the blood vasculature (65). There are four neuroimmune interfaces that keep the blood separate from the brain parenchyma: the brain blood barrier (BBB), the arachnoid epithelium (meningeal barrier), the choroid plexus (blood–CSF barrier), and the glymphatic circulatory system. These interfaces regulate the movement of ions, molecules, and cells between the brain and the blood (66). The disruption of these barriers is associated with various neurological disorders, including Alzheimer’s disease, amyotrophic lateral sclerosis, and multiple sclerosis (67). In NPSLE, the leakage of the BBB is reported to causes diffuse neuropsychiatric symptoms, such as memory deficits and depression (50, 57, 67). A surrogate marker for a loss of integrity of the BBB is the presence of serum albumin in the CSF (68, 69). A study by Hirohata et al. demonstrated a correlation between the damage of the BBB (elevated CSF serum albumin quotient + elevated CFS anti-NR2) and the presence of NPSLE compared to healthy controls (69). Although seropositivity for autoantibodies such as anti-NMDA receptor antibodies is not consistently correlated with NPSLE activity, titers of these antibodies in the CSF are higher in patients with active diffuse NPSLE than in those with focal NPSLE or non-inflammatory CNS disease (45, 46). Up to now, it seems that the intravenous application of antibodies only leads to neuropsychiatric symptoms after the disruption of the BBB (50, 70). Experimental studies indicate that an event, such as the injection of LPS, by which autoantibodies can trespass the BBB and enter the CNS is necessary to induce pathology (41, 49, 70). Another possible mechanism for how autoantibodies are produced intrathecally is a (transient) BBB dysfunction, which may enable plasma cells to access the cerebrospinal fluid and the cerebrum (71). Antibodies themselves can contribute to the leakage of the BBB. Anti-NR2 activate endothelial cells and induce the expression of adhesion molecules and cytokines (72). Anti-rib P might be also involved in the disturbance of the BBB by activating monocytes and thus promoting the production of TNF-α and IL-6 (73).

There is pre-clinical evidence that the intracerebral injection of purified NPSLE-associated antibodies in mice results in specific macroscopic as well as microscopic changes of certain brain areas (41, 57).

Additionally, various cytokines have been identified as inflammatory mediators that may play a pathogenic role and might contribute to the disruption of the BBB including IL-6, IL-8, INFα, and the TNF-like weak inducer of apoptosis (TWEAK) (74, 75). In cell culture, TWEAK signaling induced increased expression of cytokines, including IL-6 and IL-8, as well as ICAM-1 and E-selectin leading to an increased brain permeability (43, 76–79).

## Peripheral nervous system disease in SLE

In order to present an overall picture of NPSLE, we briefly summarize the involvement of the peripheral nervous system in SLE. Syndromes of the peripheral nervous system (PNS) account for less than 10% of the neurological diseases in SLE patients (80, 81). Predominant events are mono- and polyneuropathies (81).

There are various concepts for the pathogenetic mechanisms of the involvement of the PNS in SLE including neurogenic inflammation (activation of nociceptors by chemokines followed by the release of different neuropeptides such as calcitonin gene-related protein and substance P resulting in vasodilatation and increased vascular permeability) and antibody-mediated neuropathy (80, 82, 83).

Bechter hypothesized the interaction of the CFS with peripheral nerves *via* the “peripheral cerebrospinal fluid outflow pathway (PCOP),” which is already known in mammals. PCOP describes the outflow of CFS along cranial nerves and spinal nerves into the periphery (84). This might be another possible pathomechanism how CNS antigens are transported to peripheral nerves.

## Neuroimaging

Magnetic resonance imaging (MRI) and positron emission tomography (PET) represent the most commonly used tools to investigate the structure and function abnormalities in NPSLE (74, 75).

Some studies using conventional brain MRI showed white matter hyperintense lesions as well as brain atrophy in patients with SLE (76, 77). Interestingly, there were no differences between patients with or without neuropsychiatric symptoms and the number of white matter lesions did not correlate with the clinical manifestations (78).

Margo-Checa et al. investigated brain abnormalities in 325 patients with active NPSLE and showed that none of the autoantibodies measured in the serum (antiphospholipid autoantibodies, anti-dsDNA, anti-SSA, anti-SSB, anti-RNP, and anti-Sm) correlated with the presence of inflammatory-type lesions or white matter lesions. Of note, that there was a low prevalence of inflammatory-like lesions (5.8%) among all patients (85).

Taken together, conventional MRI is a good imaging method to show pathologies of the CNS, but up to now it is not an appropriate tool to differentiate between SLE with and without overt neuropsychiatric symptoms, as pathologies do not correlate with clinical manifestations nor with SLE-specific antibodies.

Diffusion tensor MRI (DTI) makes it possible to detect altered white matter microstructure, even if it appeared normal in conventional MRI. Mackay et al. studied 37 SLE patients without neuropsychiatric symptoms and could prove a correlation between decreased microstructural integrity in the white matter and increased serum levels of anti-NR2-antibody (86). Interestingly, Nystedt et al. found alterations in the white matter structure of the rostral cingulum and parts of the corpus callosum in 64 female SLE patients in comparison to healthy controls, but could not find any differences between patients with and without neuropsychiatric symptoms (87). Kozora et al. performed a longitudinal study of DTI in SLE, showing that after 18 months increased microstructural changes can be detected, but, however, these changes were not accompanied by a cognitive decline (88). In conclusion, DTI is a valid method to identify microstructural changes and to locate brain structures affected by SLE, but is not appropriate to detect NPSLE.

Moreover, the utilization of MRI makes it is possible to measure regional changes in brain metabolism in a resting as well as in a task-induced active state, which is called functional MRI. There are different methods to measure brain activity among which measuring changes in blood oxygen (blood oxygen level dependent-contrast imaging) is the most common one (89). Studies using a resting-state MRI, showed diverse abnormalities in functional connectivity of some brain regions in patients with SLE in comparison to a healthy control group (90, 91). Studies using active-state MRI demonstrated that regardless of the disease activity SLE patients are able to recruit additional pathways, suggesting a form of compensation to execute goal-directed tasks (92, 93).

Another method to evaluate regional brain metabolism is to perform fluorine-18-fluorodeoxyglucose-positron emission tomographies (FDG-PET). Most of the studies using FDG-PET imaging in SLE report a regional hypometabolism in the frontal, temporal, parietal, and parietal-occipital regions (94–96). Mackay et al. investigated the correlation between the results from FDG-PET, DTI and cognitive testing in patients with stable SLE. They revealed a resting hypermetabolism in the hippocampus, orbitofrontal cortex and posterior putamen/globus pallidus/thalamus, occipital lobe, temporal lobe, and sensorimotor cortex. Moreover, they showed, that the hypermetabolism in some of these regions is accompanied by impaired performance in cognitive testing as well as microstructural abnormalities in gray and white matter (86). In contrast to longitudinal DTI-data by Kozora et al. the FDG-PET analysis of a subgroup of 13 showed no change in the resting hypermetabolism in follow- scans (after 15 months) (86, 88). At the moment there are different findings in FDG-PET of SLE patients, suggesting different metabolism patterns that may depend on various factors including medication as well as activity and duration of the disease.

Taking everything into consideration, neuroimaging represents a non-invasive method to investigate neuroanatomical and functional abnormalities in the vivid brain of SLE patients. However, findings are not homogeneous and up to know specific syndromes cannot be associated with specific imaging results, which might be attributed to heterogeneous study groups.

## Conclusion and future perspectives

In this review we have highlighted mouse models, antibodies, and neuroimaging techniques as commonly used tools to investigate the pathogenesis of NSPLE. At the moment, most of the research results addressing this question are promising but nonetheless inhomogeneous and incomplete, which might be partially due to reasons attributable to the selection of the study group. While some studies investigated neurological abnormalities in SLE without neuropsychiatric symptoms, others focused on patients with NPSLE or even sought for pathologies in patients with specific neuropsychiatric symptoms (89–94, 96).

Neuropsychiatric systemic lupus erythematosus represents a challenge in terms of diagnosis and treatment. The EULAR recommends that treatment should depend on whether the assumed underlying pathophysiologic mechanism is either inflammatory or embolic/thrombotic/ischemic. While the use of immunosuppressants should be favored in the former, anticoagulation therapy should be chosen in the latter (97). However, the distinction between the two may not only be difficult but also insufficient as the pathophysiology of NPSLE is yet not fully understood.

One of the key pathologies found in SLE are antibodies (2). Therefore, therapy targeting B-cells is commonly used and further evolving. Besides Rituximab, Belimumab is a human monoclonal antibody which is used to decrease B-cell activity. Belimumab inhibits the biologic activity of an immunomodulatory cytokine called soluble B-lymphocyte stimulator (BLyS) (98). Schwarting et al. were able to confirm that treatment with belimumab results in a reduction of serum Anti-NR2, accompanied by an amelioration of fatigue in SLE. Interestingly, anti-dsDNA, erythrocyte sedimentation rate, complement factors, and C-reactive protein did not show significant differences (47). These findings indicate that as different antibodies/cytokine profiles are involved in the pathogenesis of specific symptoms in NPSLE, research should aim to develop targeted therapies.

In the case of Anti-NR2, we certainly think of NMDA-receptor antagonists, such as Memantine, an approved non-competitive NMDA receptor antagonist, used in the therapy of Alzheimer’s disease (99). Whereas *in vitro* studies showed that NDMA-receptor antagonists prevented neuronal damage (47, 100), a randomized, double-blind placebo-controlled trial could not detect a significant improvement in cognitive performance compared to the placebo group (101). Of note, Anti-NR2 was very infrequent in the study population.

The diagnosis of NPSLE is challenging. There is no simple test that confirms the disease. Therefore, serological testing and neuroimaging must be combined to aid in the diagnosis of neuropsychiatric involvement in SLE. It should be noted here that not every patient with serological markers or imaging abnormalities, such as white matter lesions or brain atrophy, presents clinical symptoms of NPSLE (87, 88, 44). In this context, it should be noted that none of the MRI-visible lesions is pathognomonic for SLE.

Mouse models of disease are of immense importance to give us an insight into pathogenesis of neuropsychiatric involvement in SLE. However, none of the mutations and genetic risk factors found in murine models of NPSLE commonly occur in patients with SLE (66). Nevertheless, most of the mouse models produce antibodies similar to SLE patients and exhibit important clinical manifestations, which makes it possible to study specific underlying pathways and generate therapeutic options.

## Author contributions

VT: conceptualization, methodology, visualization, and writing—original draft. MJS: supervision and writing—review and editing. JW-M: conceptualization, supervision, and writing—review and editing. All authors contributed to the article and approved the submitted version.
